# Complication after inadvertent Nd:YAG laser capsulotomy in a phakic eye

**DOI:** 10.1016/j.ajoc.2025.102468

**Published:** 2025-11-07

**Authors:** Pedro Ogata Kodama, Leonardo Luis Cassoni, Carlos Yuji Nunomura, Rodrigo Jorge

**Affiliations:** a Division of Ophthalmology, Ribeirão Preto Medical School, University of São Paulo, Ribeirão Preto, Brazil; b Medical Assistant of Sector Cornea and Cataract – Ribeirão Preto Medical School, University of São Paulo, Ribeirão Preto, Brazil; c Head of the Division of Ophthalmology, Ribeirão Preto Medical School, University of São Paulo, Ribeirão Preto, Brazil

**Keywords:** Cataract, Nd-YAG laser, Posterior capsulotomy, Posterior capsular rupture

## Abstract

**Purpose:**

To report a case of complication after inadvertent Nd:YAG laser in a phakic eye.

**Observations:**

A 65-year-old man presented with a sudden vision loss on his right eye after undergoing Nd:YAG laser for posterior capsulotomy. Best-corrected visual acuity (BCVA) was hand motion and slit-lamp examination revealed a dense posterior cataract with 3 circular holes on posterior capsule and on anterior segment OCT confirmed a posterior capsule rupture (PCR). Due to scattered fine echoes on eye ultrasound, suggesting remnants of cataract, the patient underwent phacoemulsification with posterior vitrectomy and IOL implantation in the ciliary sulcus. One day later BCVA was 20/100 and one month later improved to 20/50. Although rare, inadvertent Nd:YAG laser on phakic eye is a complication that can result in traumatic cataract, PCR and vitreous loss. These complications can be minimized through careful preoperative assessment and adherence to a strict time-out immediately before the procedure.

**Conclusion and importance:**

We describe a case of complication after inadvertent Nd:YAG laser in a phakic eye. The slit-lamp examination and anterior OCT revealed a traumatic cataract and a PCR. These complications were successfully managed with combined phacoemulsification and pars plana vitrectomy, resulting in improved BCVA postoperatively. The case highlights the importance of preoperative time-out to prevent a rare complication after Nd:YAG laser.

## Highlights

• Inadvertent Nd:YAG laser capsulotomy in a phakic eye resulted in traumatic cataract and posterior capsule rupture.
• Successful management was achieved with combined phacoemulsification and pars plana vitrectomy with sulcus IOL implant.
• Case underscores the importance of the Universal Protocol and “always event” in preventing “never events” in ophthalmology.

## 1 Introduction

Posterior capsulotomy using neodymium:yttrium–aluminum–garnet (Nd: YAG) laser is a common and widely used procedure for the management of posterior capsule opacification (PCO). It can be performed in the office and usually provides excellent outcomes, with improved visual acuity and contrast sensitivity. Although generally considered a straightforward procedure, it may lead to complications such as elevated intraocular pressure, intraocular lens (IOL) displacement, ocular inflammation, cystoid macular edema, retinal tears, and retinal detachment.^1^^,^^2^ Another rare complication is when inadvertently performed in phakic eyes, causing traumatic cataract, posterior capsule rupture (PCR), and vitreous loss.^2^

Careful preoperative assessment of pseudophakic eyes, accurate PCO classification, and strict time-out protocols make inadvertent posterior capsulotomy an uncommon event. Only two cases have been reported in the literature.^3^^,^^4^ We describe the surgical management of a traumatic cataract complicated by posterior capsule rupture after inadvertent Nd:YAG laser application in a phakic eye.

## 2 Case report

A 65-year-old man with past ocular history of high myopia and blindness on his right eye (OD) due to retinal detachment previously undergone phacoemulsification and pars plana vitrectomy (PPV). He presented to emergency department complaining of sudden vision loss in his left eye (OS) right after an ocular laser procedure. One week earlier, the patient had undergone posterior capsulotomy with Nd:YAG laser at another service.

On ophthalmological examination best visual acuity (BCVA) was hand motion in OD and counting fingers in OS. Slit-lamp biomicroscopy OD revealed a clear cornea, well-centered IOL and PCO 2/4+, while OS revealed a dense posterior cataract with three circular holes on posterior capsule approximately 2 mm diameter (blue arrows; Fig. 1a). The anterior segment OCT demonstrated a PCR as well (green arrow; Fig. 1b). The OD fundoscopy revealed an attached retina with macular atrophy and was impossible on OS due to media opacity. A B-scan ocular ultrasound was then performed and revealed scattered fine echoes, suggesting remnants of cataract. The intraocular pressure was normal in both eyes. Based on the clinical findings, a combined phacoemulsification and PPV was proposed for OS. This management was discussed with the patient, who was counseled about the complexity of his case and the guarded visual prognosis.

**Fig. 1a:**
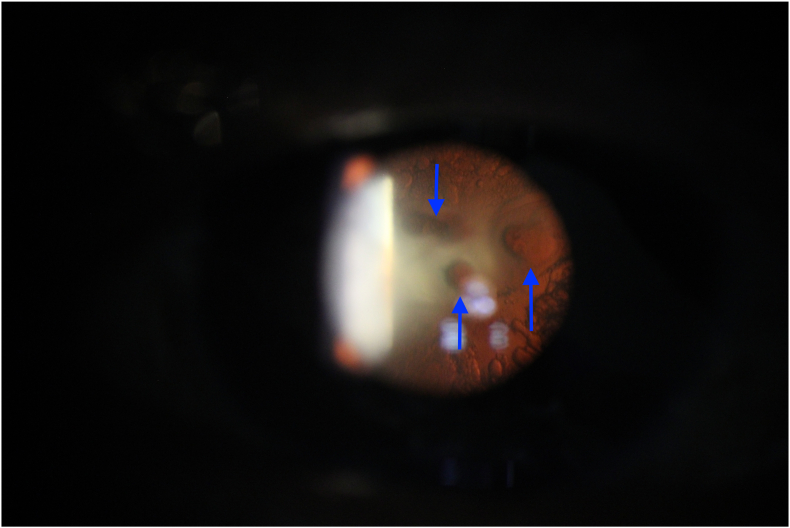
Slit lamp retroillumination photography demonstrating three circular holes on posterior capsule approximately 2mm diameter (blue arrows).

**Fig. 1b:**
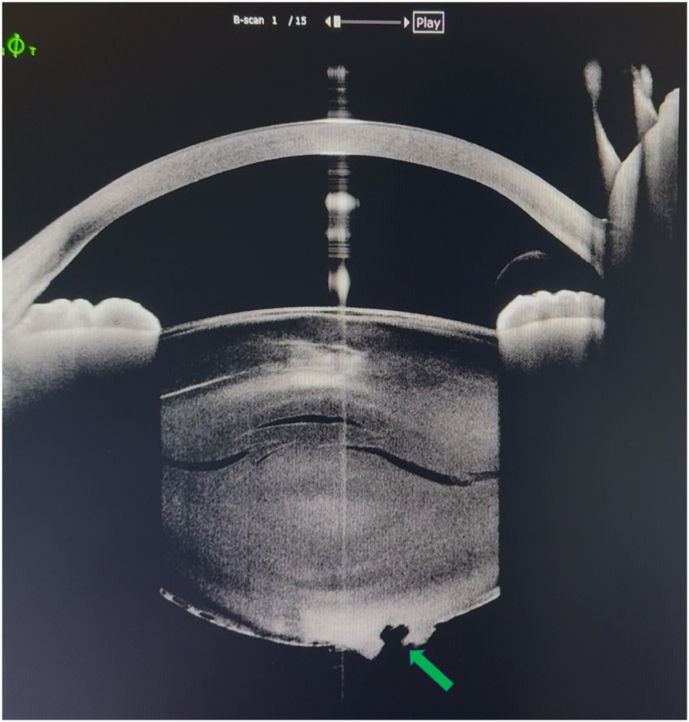
Anterior segment OCT with a posterior capsule rupture pointed with a green arrow.

During surgery, PCR was observed in three circular holes on the posterior capsule, approximately 2mm in diameter each with smooth regular borders and a dense opacified margins, consistent with a typical appearance of laser-induced marks. After capsulorhexis, hydrodelineation was performed, followed by nucleus fracture. The fragments were then aspirated with minimal phacoemulsification energy. The remaining cortex associated with fibrosis of the posterior subcapsular cataract (PSC) was removed with visco-dissection technique. Due to capsular instability, a three-pieces Acrysoft MA60AC (Alcon Laboratories, USA) IOL was implanted in the ciliary sulcus with optic capture in the anterior rhexis. PPV was subsequently performed to remove all cortical debris within the vitreous cavity. The procedure ended with a detailed retinal examination visualization and 360° endolaser photocoagulation, without complications. In our service, 360° endolaser photocoagulation is routinely performed to reduce peripheral retinal breaks and postoperative traction, especially in patients with history of retinal detachment in the contralateral eye.

During the postoperative follow-up, the patient was treated with a regimen of topical corticosteroids and prophylactic antibiotics. On day one he presented visual acuity of 20/100 and corneal edema 1+. The IOL was stable in the sulcus (Fig. 2a) and with optic capture in the anterior rhexis (Fig. 2b). The retina was attached without cortical remnants. After one month, there was a complete improvement of the corneal edema and the BCVA was 20/50 with a refraction of +2.00 -3.75 X 135°.

**Fig. 2a:**
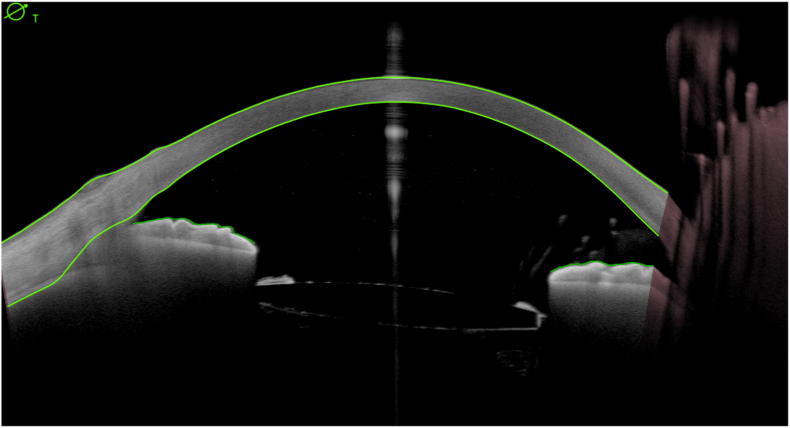
Anterior segment OCT with an IOL implanted in ciliary sulcus.

**Fig. 2b:**
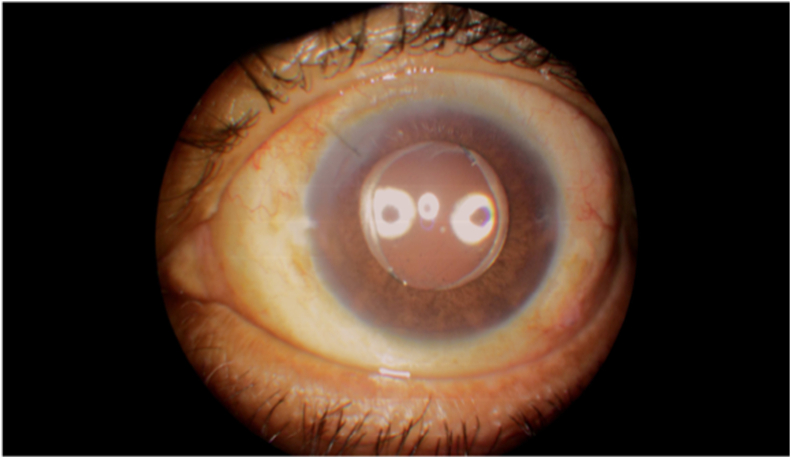
Anterior segment photography on seventh day after surgery, showing a well-centered IOL with optic capture in the anterior rhexis.

## 3 Discussion

Nd:YAG laser capsulotomy is considered the standard treatment for PCO. It is a noninvasive and generally safe procedure; however, it is not without risk of complications.^1^ The present case reports a rare complication: an inadvertent Nd:YAG laser in a phakic eye. This event could be potentially devastating, as it may result in traumatic cataract, PCR and vitreous loss.^2^ The presence of PCR and possible remnants of cataract in vitreous cavity in this case required a phacoemulsification combined with PPV. The vitrectomy was indicated due to possible remnants of cataract in vitreous cavity revealed on B-scan ultrasound and to ensure complete removal of opacified material and vitreous traction.

During surgery, aspiration of the cortical remnants along with the subcapsular cataract fibrosis was challenging, particularly due to concern of avoiding further of enlargement of the posterior capsule tear. Therefore, the viscodissection technique was essential, reducing traction forces on the posterior capsule.^5^ After phacoemulsification, the three-pieces IOL was implanted in the ciliary sulcus with optic capture, providing good IOL stability even with extensive PCR and capsular instability.^6^^,^^7^ Postoperatively, the patient achieved significant visual improvement, with stable retinal attachment during follow-up.

Few similar cases have been described in the literature. Ibrahim et al.^3^ described a patient undergoing phacoemulsification who, early in the surgery, experienced cataract fragments falling into the vitreous cavity. Immediately afterward, posterior pars plana vitrectomy was required to remove the vitreous fragments. A review of the medical records revealed that the patient underwent Nd:YAG laser therapy after being diagnosed with PCO rather than dense PSC. Another case described by Moshirfar, Majid et al.^4^ also reports the inadvertent application of laser therapy to a phakic eye after the same mistake: confusing PCO with PSC.

In this case, the Nd:Yag laser was mistakenly performed on the wrong eye. The pseudophakic eye was OD and had a PCO 2+/4+. This error most likely occurred due to confusion between PCO and PSC. It is important to recognize conditions that may increase the likelihood of such misinterpretation. When the examination is performed under high magnification and using a contact lens, the posterior lens surface may appear as an opaque capsule, particularly when the mydriasis is not complete. Other contributing factors include inadequate preoperative evaluation, incomplete or inaccurate medical documentation and misinterpretation of patient records.

All these 3 reports emphasize that careful preoperative assessment of pseudophakic eyes, accurate PCO classification, and strict time-out protocols are essential to minimize the risk of inadvertent posterior capsulotomy in a phakic eye. Moshirfar, Majid et al.^4^ also highlights the Universal Protocol introduced by the Joint Commission in 2004. This protocol was implemented to prevent wrong-site, wrong-person and wrong-procedure surgical errors. It also extends to clinical settings outside the operating room, for any invasive procedure that require patient consent.^8^ The Universal Protocol consists of three steps: pre-procedure verification process; mark the procedure site; and perform a time-out. The first consists of before the procedure, the healthcare team conducts a thorough verification to confirm the correct patient, surgical site, and procedure. This is done using patient records, consent forms, and diagnostic results. The second step is to do a clearly mark on the site of the procedure, ensuring visibility. The last step is to confirm once again with all members the correct patient, site and surgery right before the procedure.^9^ Despite all efforts and widespread implementation of Universal Protocol, these events have still been reported.

The National Quality Forum (NQF) introduced in 2002, in the United States, the term “never events” referring to 27 serious medical errors that are identifiable, preventable and of concern for both patients and healthcare providers. This list was updated in 2006 with one additional event and organized into six categories,^10^ including surgical procedures (Table 1). Our report of an inadvertent laser capsulotomy in a phakic eye aligns with this concept of a “never event” specifically within the category of surgery performed on the wrong body part or the wrong surgical procedure performed on a patient.

**Table 1:** Serious Reportable Events (“never events”) in healthcare, as defined by the National Quality Forum (NQF – A Consensus Report, update 2011).

Surgical or Invasive Procedure Events	
A.	Surgery or other invasive procedure performed on the wrong site
B.	Surgery or other invasive procedure performed on the wrong patient
C.	Wrong surgical or other invasive procedure performed on a patient
D.	Unintended retention of a foreign object in a patient after surgery or other invasive procedure
E.	Intraoperative or immediately postoperative/postprocedure death in an ASA Class 1 patient

At the same time, this adverse event also illustrates the complementary concept of “always events”. These are evidence-based practices that must reliably occur in all patient care to promote safety and ensure high-quality outcomes.^11^

This case underscores how PSC may mimic PCO under certain optical conditions. Reinforcing adherence to standardized time-out and verification checklists—core components of the Universal Protocol—remains essential to prevent such avoidable and potentially vision-threatening errors.

In the context of laser procedures, we emphasize the critical importance of a thorough preoperative assessment, accurate evaluation of the lens status and the patient's surgical history and a strict time-out protocol. These steps not only help prevent a “never-event” but also strengthen patient engagement and trust in care.

## 4 Conclusion

This case highlights that preventing “never events” in ophthalmology relies not only on technical expertise but also on the consistent application of “always events”, reinforcing a culture of patient safety and trust in every procedure. Fortunately, despite experiencing a never event, our patient achieved a good improvement in BCVA after surgical management.

## CRediT authorship contribution statement

**Pedro Ogata Kodama:** Writing – review & editing, Writing – original draft, Investigation, Conceptualization. **Leonardo Luis Cassoni:** Writing – review & editing, Writing – original draft, Investigation, Conceptualization. **Carlos Yuji Nunomura:** Writing – review & editing, Supervision, Investigation. **Rodrigo Jorge:** Writing – review & editing, Validation, Supervision, Investigation.

## Patient consent

Consent to publish this case report has been obtained from the patient in writing.

## Authorship

All author attest that they meet the current ICMJE criteria for Authorship.

## Funding sources

This research did not receive any specific grant from funding agencies in the public, commercial, or not-for-profit sectors.

## Declaration of competing interest

The authors declare that they have no known competing financial interests or personal relationships that could have appeared to influence the work reported in this paper.
